# DNA replication timing influences gene expression level

**DOI:** 10.1083/jcb.201701061

**Published:** 2017-07-03

**Authors:** Carolin A. Müller, Conrad A. Nieduszynski

**Affiliations:** Sir William Dunn School of Pathology, University of Oxford, Oxford, England, UK

## Abstract

Eukaryotic genomes are replicated in a reproducible temporal order; however, the physiological significance is poorly understood. We compared replication timing in divergent yeast species and identified genomic features with conserved replication times. Histone genes were among the earliest replicating loci in all species. We specifically delayed the replication of *HTA1*-*HTB1* and discovered that this halved the expression of these histone genes. Finally, we showed that histone and cell cycle genes in general are exempt from Rtt109-dependent dosage compensation, suggesting the existence of pathways excluding specific loci from dosage compensation mechanisms. Thus, we have uncovered one of the first physiological requirements for regulated replication time and demonstrated a direct link between replication timing and gene expression.

## Abstract (precis)

Eukaryotic genomes are replicated in a reproducible temporal order whose physiological significance is poorly understood. Müller and Nieduszynski compare the temporal order of genome replication in phylogenetically diverse yeast species and identify genes for which conserved replication timing contributes to maximal expression.

## Introduction

Eukaryotic genomes replicate in a characteristic and reproducible temporal order dictated by the location and activity of replication origins. Replication timing correlates with gene expression, chromatin state, GC content, and subnuclear structure (Rhind and Gilbert, 2013). These correlations might reflect the variable accessibility of origins to a limited pool of initiation factors within different contexts (Mantiero et al., 2011; Tanaka et al., 2011; Collart et al., 2013; Köhler et al., 2016). However, at least a subset of replication origins is regulated by direct recruitment of activating factors (e.g., Dbf4 recruitment to centromere-proximal origins; Natsume et al., 2013) or inhibitory factors (e.g., Rif1 to telomere-proximal origins; Hayano et al., 2012; Sridhar et al., 2014). Overall, the temporal order of genome replication is sufficiently characteristic to allow identification of cell types (Ryba et al., 2010). Furthermore, comparative genomic analyses have revealed that the temporal order of genome replication is conserved between closely related species of yeast or mammals (Yaffe et al., 2010; Müller and Nieduszynski, 2012), although specific loci may be replicated at different times because of variations in cis-acting elements (Müller and Nieduszynski, 2012; Koren et al., 2014). Therefore, the broad conservation of replication time is consistent with physiological requirements for regulation of replication timing; however, few such loci have been identified to date.

An appropriate number and distribution of replication initiation sites is essential for genome stability. Global deregulation of origin activity leads to DNA damage and genome instability. For example, massive overexpression of rate-limiting factors allows excessive origin activation, which results in DNA damage and cell death (Mantiero et al., 2011; Tanaka et al., 2011). Conversely, inactivation of many adjacent origins or a dramatic reduction in the concentration of licensing or firing factors increases the likelihood of incomplete genome replication (Theis et al., 2010; Alver et al., 2014). However, inactivation of single or few origins has rarely been observed to affect cell fitness in yeast (Hawkins et al., 2013). An exception is the inactivation of centromere-proximal origins, such that centromere replication is delayed, which results in a chromosome loss phenotype (Natsume et al., 2013). Therefore, although global control over the rate of origin activation is crucial to prevent DNA damage, there is only limited understanding of the requirements for local temporal control.

The high demand for histones during S phase is met by multiple copies of each gene and cell cycle regulation of expression and transcript stability; this helps ensure that histone synthesis is tightly coupled with the requirement to package newly replicated DNA (Hereford et al., 1981; Kurat et al., 2014). For example, global inhibition of DNA replication leads to a significant reduction in histone gene transcript levels (Hereford et al., 1981; Omberg et al., 2009). In *Saccharomyces cerevisiae*, bidirectional promoters drive expression of histone gene pairs that encode dimerizing histones. In addition, histone gene pairs are frequently closely positioned to replication origins. For example, the *HTA1*-*HTB1* gene pair is associated with an origin in *S. cerevisiae*, *Lachancea kluyveri*, *Kluyveromyces lactis*, and *Lachancea waltii* (Di Rienzi et al., 2012). In *S. cerevisiae*, linkage between histone genes and an origin is not required for appropriate histone expression (Osley et al., 1986).

Here, we have compared the temporal order of genome replication in phylogenetically diverse yeast species and identified genomic features with conserved replication timing. We discovered that many genes possess a conserved replication time, consistent with a physiological requirement to replicate at a particular time during S phase. As an example, we demonstrate that early replication of histone genes is required for their maximal expression in S phase.

## Results

### Temporal order of genome replication in diverse species

**Figure 1.**
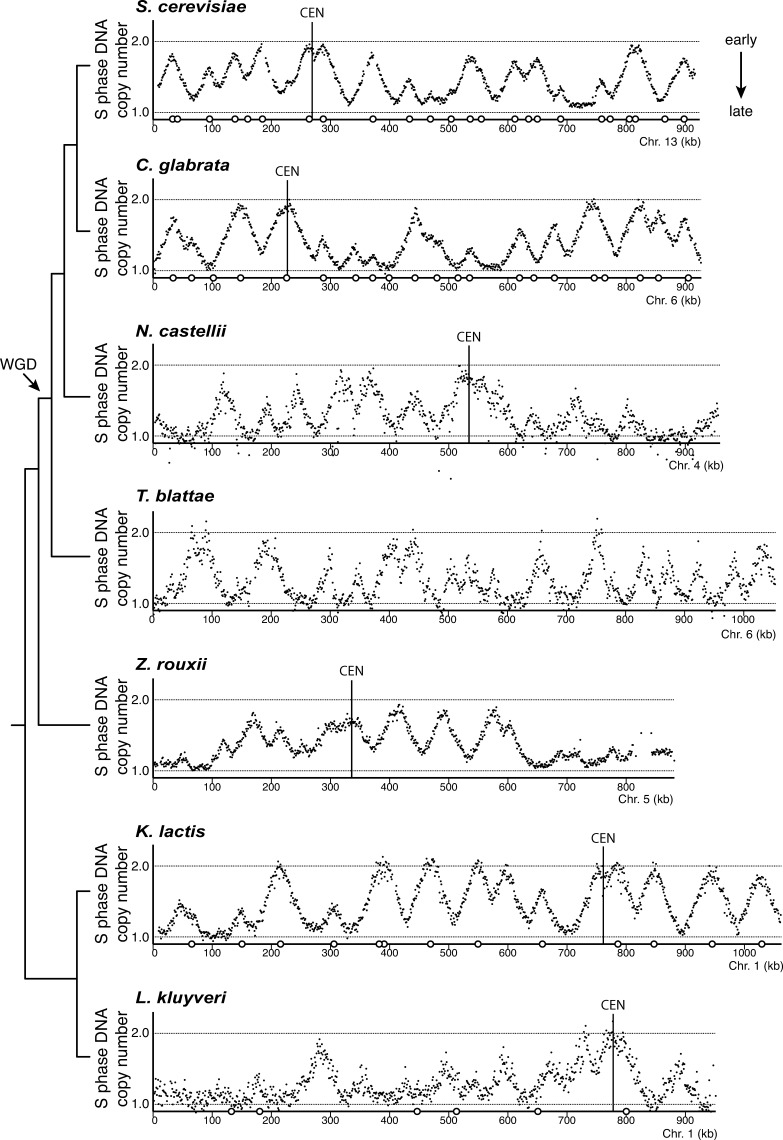
**The dynamics of genome replication in seven divergent yeast species.** Each profile shows an example chromosome, with the position on the *x* axis and the normalized relative copy number (as a proxy for replication time) on the *y* axis. Previously annotated origins (Liachko et al., 2010, 2011; Siow et al., 2012; Descorps-Declère et al., 2015) are shown as circles on the *x* axis and centromeres as vertical lines. The phylogenetic relationship between the species and the WGD event are indicated on the left. *Z. rouxii*, *Zygosaccharomyces rouxii*.

We hypothesized that replicating particular genomic features at specific times during S phase may be physiologically important, and then the replication time of such features would be evolutionarily conserved. To directly test this hypothesis, we examined the temporal order of genome replication in seven divergent budding yeast species (Fig. 1). These species represent the breadth of phylogenetic divergence within budding yeasts, spanning differences in amino acid identity comparable to that between mammals and fish (Dujon, 2006). Previously, we have shown that genome replication timings are remarkably similar between closely related species with extensive synteny (Müller and Nieduszynski, 2012). Therefore, for our approach to be valid, it was necessary that the species divergence was sufficient to break synteny between genes as well as between genes and replication origins. Comparisons between *S. cerevisiae* ohnologs (paralogs formed by whole genome duplication [WGD]) provide a measure of replication timing conservation, because they represent an evolutionary distance comparable to or less than that between our species comparisons. The majority of *S. cerevisiae* ohnologs do not have similar replication times (Fig. S1 A), and a previous study found no conservation in replication origin location after the WGD (Di Rienzi et al., 2012). Furthermore, there was substantial loss of synteny between the seven species we selected: the mean size of synteny blocks between these species (∼20 kb; Fischer et al., 2006) is several times smaller than the distance between chromosomally active replication origins (∼75 kb). Previous studies identified little conservation in origin location between some of our selected species (Liachko et al., 2010; Agier et al., 2013). In addition, the evolutionary distances covered by our selected species are comparable to a pairwise comparison between *S. cerevisiae* and *L. waltii* that found no evidence for global conservation of replication timing (Di Rienzi et al., 2012). Therefore, we were confident that our selected species were sufficiently divergent to reveal physiological requirements for regulation of replication timing.

For each species, the relative replication timing was determined by sort-seq analysis (Müller et al., 2014). In each species, the replication timing profile (Fig. 1) broadly resembled those from *S. cerevisiae*, with clearly defined peaks denoting replication origin locations and clusters of early- or late-activating origins distributed along each chromosome. Centromeres were among the earliest replicated regions of every chromosome in each species, whereas telomeres tended to be late replicating; this is consistent with expectations from *S. cerevisiae* (McCarroll and Fangman, 1988) and other species. In addition, our replication profiles were in close agreement with those independently determined for *L. kluyveri* and *Candida glabrata* (Agier et al., 2013; Descorps-Declère et al., 2015). Finally, peaks in our replication profiles colocalized with the limited number of previously reported sites that support plasmid replication (Fig. 1). Therefore, these comparisons offered strong validation of our data. Together with the use of a common experimental approach, our data provided the basis for a comparative analysis of replication timing in diverse species.

### Identification of genomic features with evolutionarily conserved replication timing

**Figure 2.**
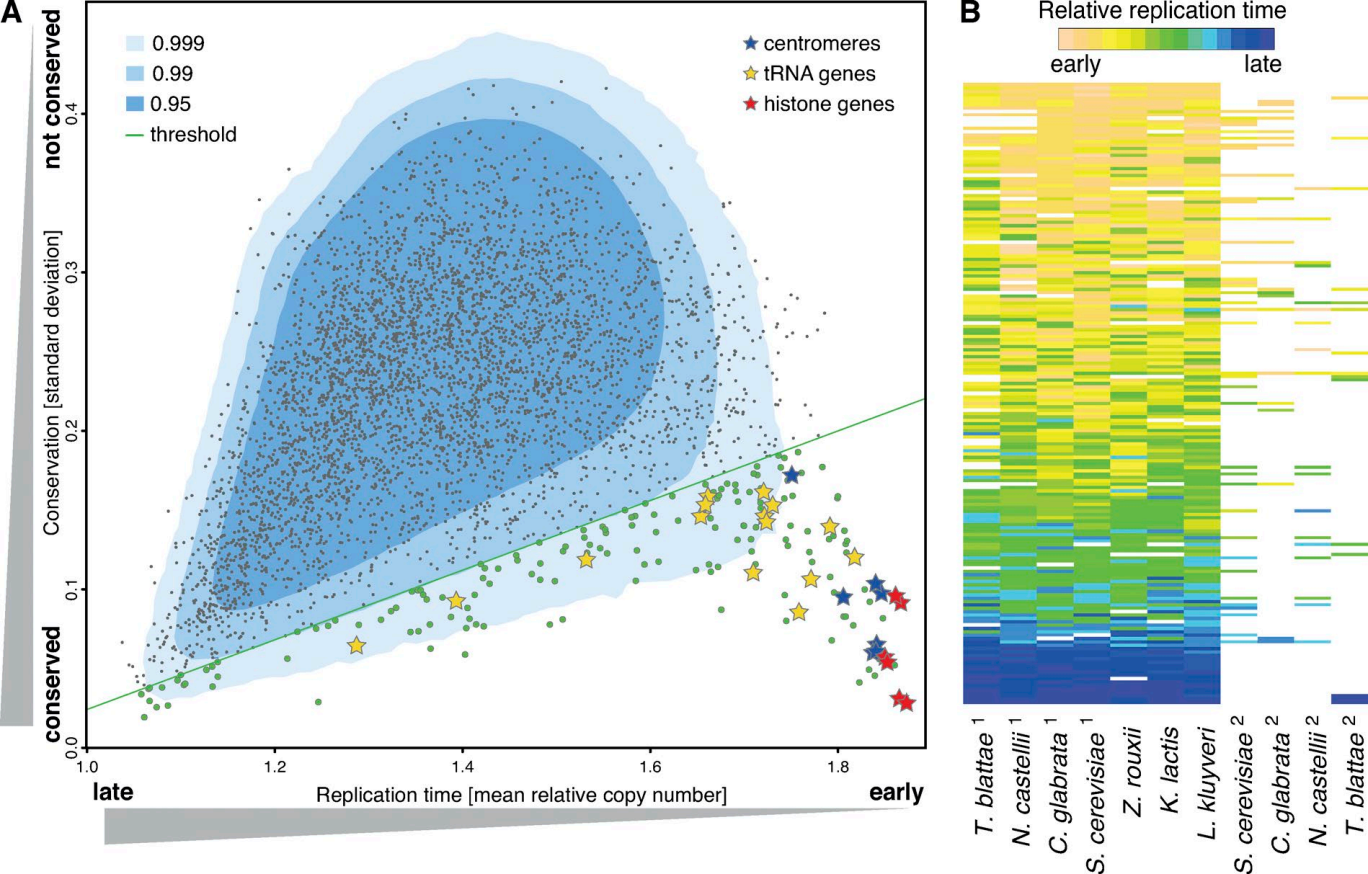
**Identification of genomic elements with evolutionarily conserved replication time.** (A) Comparison of the degree of conservation in replication time between yeast species for ancestrally related genetic elements (Byrne and Wolfe, 2005). The *x* axis is a proxy for the mean replication time between species; the *y* axis is a proxy for degree of evolutionary conservation in replication time. Points represent experimental data (*n* = 4,616); the blue surfaces represent the proportion of data (0.95, 0.99, and 0.999) from random simulations. The green diagonal line represents the threshold below which the conservation was considered to be significant. Three classes of genetic elements with conserved replication times are indicated. (B) Visualization of replication timing data for those ancestrally related genetic elements with a conserved replication time (*n* = 185). Rows represent genetic elements; columns indicate the species. *Z. rouxii*, *Zygosaccharomyces rouxii*.

To compare the replication time of genetic elements, we assigned their homologs and ohnologs based on previously determined common ancestry (Gordon et al., 2009). Then, the relative replication time of every element from each species was assigned from our genome-wide data. For subsequent analyses, we retained only those elements, including genes and centromeres, for which six or more replication timing values were available. These excluded genetic elements that were annotated in only a minority of species. In total, we considered 4,616 ancestral elements that represented 5,220 *S. cerevisiae* genes/centromeres and included 76% of *S. cerevisiae* protein-encoding genes. The majority of ancestral elements had a single value from each of the seven species (Fig. S1 B). For each element, the available replication timing values were used to calculate the cross-species mean replication time and SD. A low SD represents low variation in the replication time between the species and therefore serves as a proxy for conservation of replication time. We compared the observed level of evolutionary conservation in replication time with a random model (Fig. 2 A). In both the observed and model data, there was a bias toward lower SDs for late-replicating elements. This is likely to be a consequence of there being more late- than early-replicating loci in the data from each species. We selected a threshold that excluded >99% of the model data (Fig. 2 A, green line); at that threshold, 185 ancestral elements were retained (Fig. 2 B and Table S2). By comparison, 3,000 iterations of the model had a mean of 19.6 and a maximum of 34 elements below the threshold (Fig. S1 C). This finding is consistent with an evolutionary selective pressure to maintain replication time for a subset of genetic elements. Because of the inclusion of ohnologs from the WGD event, these 185 ancestral elements correspond to 221 *S. cerevisiae* elements (182 protein-encoding genes, 25 tRNA genes, and 14 centromeres; Table S3 A).

We sought to confirm that the species we analyzed had sufficient breakdown in genetic linkage to independently test the conservation of replication time of genetic elements. First, for the 221 *S. cerevisiae* elements with a conserved replication time, we found that neighboring elements displayed low conservation in replication time, comparable to that seen for all elements (Fig. S1 D). Second, we found that these 221 elements were present within 142 genomic clusters, the majority of which (100) contained a single gene (Fig. S1 E). In the remaining 42 clusters, we anticipated at least one element per cluster to be under selective pressure to retain its replication time. Indeed, there are clear examples of clusters with more than one element likely to be under such selective pressure (for example, *CEN2* adjacent to *HTA2* and *HTB2*). Therefore, the majority of the 221 *S. cerevisiae* elements are likely to have been subject to an evolutionary selective pressure to conserve the replication time, consistent with many independent physiological requirements for regulated replication timing.

To further explore the 221 *S. cerevisiae* elements with conserved replication times, we looked for common functional annotations. The three most significantly enriched elements were centromeres (P = 2.2 × 10^−16^), tRNA genes (P = 5.7 × 10^−8^), and histone genes (P = 2.6 × 10^−5^; Table S3). The identification of centromeres validated our approach, because we have previously discovered that regulated centromere replication time contributed to stable chromosome inheritance (Natsume et al., 2013). However, centromeres are known to repress local recombination and consequently limit the evolutionary breakdown of centromere-proximal linkage (Vincenten et al., 2015). Therefore, it is possible that we identified some genes as a result of centromere linkage rather than an independent physiological requirement for regulated replication timing. To assess this, we analyzed published data in which the centromere-dependent early replication time was specifically abrogated (Natsume et al., 2013) and identified 39 (of 182 protein-encoding and 25 tRNA) genes with a significantly altered replication time. It is possible that some of these 39 genes do not have a conserved replication time independent of centromeres. Notably, some centromeres remain early replicating despite abrogation of Dbf4-dependent kinase recruitment to the kinetochore. Therefore, there are clearly additional mechanisms that can give rise to early centromere replication, perhaps as a consequence of linked elements having their own requirements for early replication.

The second most significantly enriched feature was tRNA genes. A linkage between tRNA genes and replication origins has previously been reported in *S. cerevisiae* (Wyrick et al., 2001). We confirmed a comparable linkage in the other six species (unpublished data), thus accounting for the observed conservation of replication time. We discovered a small but significant bias toward codirectionality of tRNA transcription and replication (Fig. S1 F). This is consistent with a level of genome organization that minimizes conflicts between tRNA transcription and replication, such as fork stalling events (Deshpande and Newlon, 1996; Osmundson et al., 2017). Surprisingly, we did not observe a general co-orientation of transcription and replication across the genome, even for highly expressed protein-encoding genes (unpublished data). Therefore, the repetitive nature of tRNA genes may impose a greater requirement for codirectionality of their transcription and replication (relative to protein-coding genes) to prevent potentially erroneous recombination.

### Early DNA replication contributes to maximal histone gene expression

**Figure 3.**
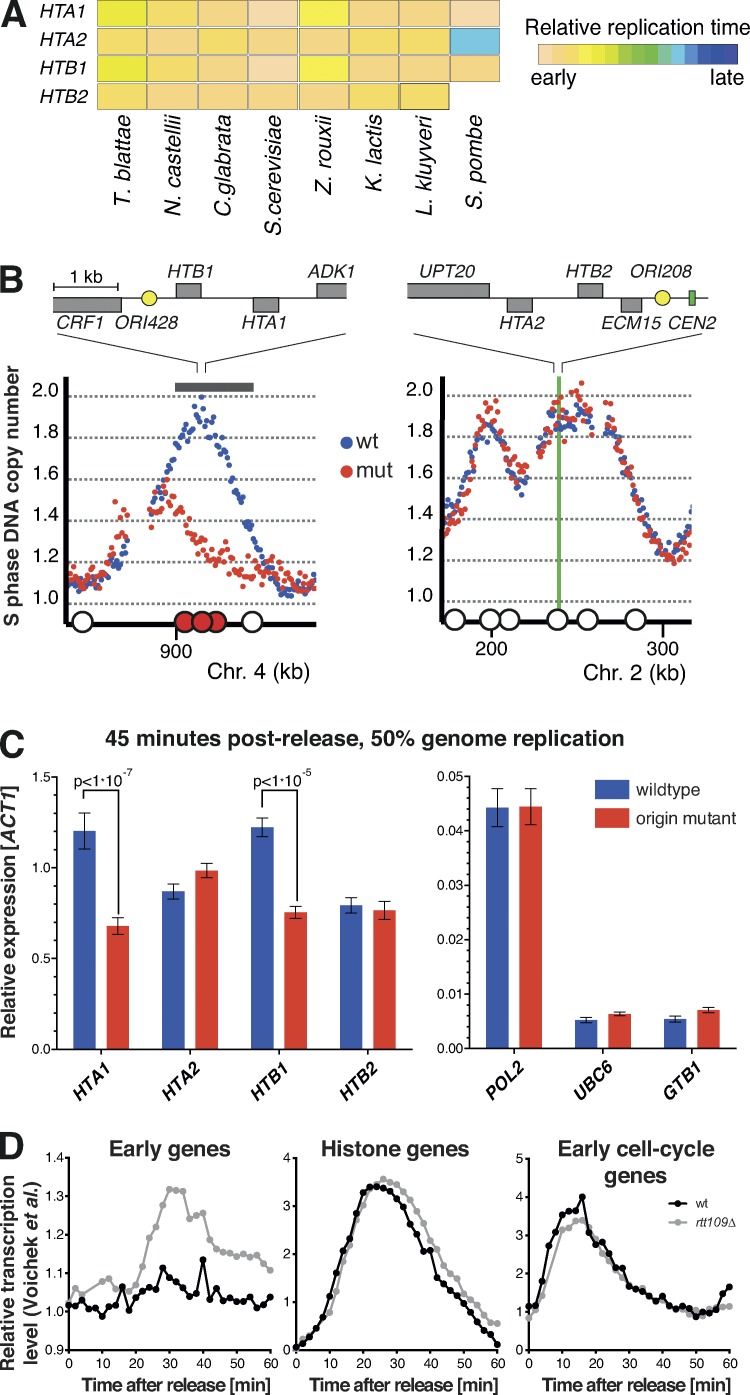
**Gene expression control at the level of DNA replication timing.** (A) Genes encoding H2A and H2B are early replicating in divergent yeast species. (B) Inactivation of three origins (large filled red circles) significantly delayed replication of the region containing *HTA1* and *HTB1* (gray bar; P ≤ 0.001). Relative replication timing for regions flanking histone genes on chromosomes 2 and 4 for wild-type (blue) and origin mutant (red) cells. (C) RT-qPCR measurement of transcript levels in mid-S phase demonstrated that delayed DNA replication resulted in a significant reduction in *HTA1* and *HTB1* expression, but no change for control genes (two-tailed *t* test). Error bars represent ± SD between five technical repeats (biological repeat in Fig. S3). (D) Cell cycle–regulated genes are excluded from dosage compensation. Comparison of early replicating housekeeping (left), histone (middle), and cell cycle–regulated (right; excluding histone) gene transcript levels during S phase in wild-type and *rtt109Δ* cells (RNA-seq; Voichek et al., 2016). *Z. rouxii*, *Zygosaccharomyces rouxii*.

Histone genes were the most significantly conserved set of protein-encoding genes discovered in our screen. For example, the four genes encoding H2A and H2B are replicated early in all species analyzed, as well as in *Schizosaccharomyces pombe* (Fig. 3 A; Daigaku et al., 2015). We sought to test the hypothesis that replication early in S phase contributes to maximal histone gene expression via doubling of gene copy number (Di Rienzi et al., 2012). A prediction of this hypothesis is that a delay in replication time would result in reduced transcript levels during S phase. *S. cerevisiae* offers a unique experimental system in which the replication time of a locus can be delayed by inactivation of nearby replication origins (Hawkins et al., 2013). We inactivated three origins proximal to *HTA1*–*HTB1* to delay replication of this gene pair. Genome-wide replication timing analysis confirmed a substantial and specific delay in replication of the region containing *HTA1*–*HTB1* (P ≤ 0.001), from the very start to the last quarter of S phase (Fig. 3 B). The rest of the genome, including the other H2A and H2B gene pair (*HTA2*–*HTB2*), showed no difference in replication timing between wild-type and origin mutant strains (Fig. 3 B).

Next, we tested whether delayed gene replication resulted in altered expression. Wild-type and mutant cells were arrested and released synchronously into S phase. We observed no change in the dynamics of cell cycle progression as a consequence of delayed histone gene replication (Fig. 3 C). Transcript levels were determined for histone and control genes at multiple time points through S phase. (None of the control genes were identified in our screen for elements with an evolutionarily conserved replication time.) The expression of control genes was not reduced in the origin mutation strain, including for 10 genes within the replication-delayed region (Fig. 3 C and Fig. S2, B and C). As a further control, we analyzed transcript levels of a cell cycle–regulated gene (*POL2*) for which the replication time was unaltered. As anticipated, the *POL2* transcript levels increased through S phase with similar kinetics for both strains (Fig. S2 B).

Next, we examined mRNA abundance of H2A- and H2B-encoding histone genes (*HTA1*, *HTB1*, *HTA2*, and *HTB2*). Transcript levels increased upon entry into S phase, peaking at 45 min (Fig. S2 D); this is consistent with previous studies (Eriksson et al., 2012; Kurat et al., 2014). Because histone mRNA is degraded in each cell cycle, these experiments are detecting de novo synthesis. We then focused on the 45-min time point, when 50% of the genome is replicated, because this is the point at which the delay in replication in the origin mutant strain would be anticipated to give a maximal difference in gene copy number. The *HTA2* and *HTB2* gene pair served as a control (for no change in replication time), and we detected no difference in transcript level. However, for both *HTA1* and *HTB1*, we observed a reduction in transcript level in the origin mutant to approximately half the wild-type levels (Fig. 3 C). This reduction in transcript levels for the replication-delayed histone genes was significant (P < 10^−5^, *t* test), it was not dependent on the gene used to normalize expression levels, it was clearly observable at other mid-S phase time points, and it was seen in a biological replicate (Fig. S3, A and B). Given that there are additional, near-identical gene copies, we anticipate modest reductions in histone protein levels. This is consistent with the wild-type and histone-delayed strains having near identical S-phase kinetics. In addition, because we observed a timely induction of *HTA1* and *HTB1* gene expression, even when replicated late in S phase (Fig. S2 D), we can exclude a direct role for DNA replication in inducing histone gene transcription. Furthermore, our findings cannot be explained by previously reported mechanisms (Hereford et al., 1981; Omberg et al., 2009) that link global DNA replication and histone supply, because we have not altered global DNA replication or cell cycle dynamics (Fig. S2 A). Therefore, we propose a novel mechanism whereby early replication contributes to maximal histone gene expression via a doubling in gene dosage.

Recently, it has been proposed that an Rtt109-dependent mechanism of dosage compensation down-regulates the expression of newly replicated genes (Voichek et al., 2016). This mechanism helps ensure constant levels of gene expression irrespective of gene replication time (Fig. 3 D). In contrast, we found that histone gene expression levels are influenced by replication time (Fig. 3 D) and are not subject to Rtt109-dependent repression (Fig. 3 D). Finally, we extended our analysis of the Voichek et al. (2016) *rtt109Δ* data and discovered that cell cycle–regulated genes in general are not subject to the Rtt109-dependent mechanism (Fig. 3 D). This raises the intriguing possibility that there are as-yet-undiscovered pathways that exclude certain genes from dosage compensation mechanisms.

During mammalian development, there are characteristic changes in the replication timing of certain genomic regions that correlate with changes in gene expression (Rhind and Gilbert, 2013). Furthermore, there are specific and reproducible changes in replication timing at early stages of carcinogenesis (Ryba et al., 2012). Although transcriptional activation can advance replication time in mammalian cells (Therizols et al., 2014), it remains unknown whether the opposite is true. Therefore, our finding in yeast, that changes in replication time can directly influence gene expression, raises the prospect that similar direct links may exist in mammalian cells. Although a dosage compensation mechanism has been described in mammalian cells (Yunger et al., 2010), we show here that such a mechanism may not apply to certain genes. It is therefore possible that the changes in replication time during carcinogenesis could affect gene expression and potentially contribute to disease progression.

## Materials and methods

### Yeast genetics and molecular biology

The origin mutant strain CAY583 was created by sequential inactivation of three origins. First, *ARS427.5* was deleted by transferring the *SPR28::KanMX* deletion cassette from the gene-deletion collection strain into a W303 wild-type background (T7107; Natsume et al., 2013). Next, *ARS429* was deleted by insertion of the *TRP1* marker, amplified from YDpW. The *spr28::KanMX*, *ARS429::TRP1* strain was diploidized by transient HO expression. Finally, *ARS428* was inactivated by a 4-bp mutation using a two-step “pop-in and pop-out” method (Struhl, 1983). In brief, *ARS428* was amplified as two overlapping fragments, with one of the primers featuring a 4-bp mismatch, creating an AgeI site in the ACS. The two fragments were combined by fusion PCR, cloned into pRS306 to generate pCA252 (Sikorski and Hieter, 1989), and verified by Sanger sequencing. The diploid *spr28::KanMX*, *ARS429::TRP1* strain was transformed with linearized pCA252. Transformants were selected on Ura dropout plates and then propagated on rich media to induce loop-out of the plasmid backbone, including the endogenous *ARS429*. Loop-out events were identified by replica plating onto 5-fluoroorotic acid and then verified by PCR and AgeI digestion.

### Generating replication timing profiles

Replication timing was determined by sort-seq analysis as described previously (Müller et al., 2014). In brief, replicating and nonreplicating cells were enriched from asynchronously growing cultures by FACS based on DNA content. Genomic DNA was extracted and sequenced to high depth (>4,000 mapped reads/kb; Table S1) to measure relative DNA copy number as a proxy for replication time. This was plotted by genomic coordinate to give replication timing profiles.

Library preparation and sequencing were performed according to Illumina instructions. Sequencing reads were mapped to the reference genome for each organism (using the Stampy package). Samtools “view” was used to filter reads, retaining uniquely mapped reads with each segment properly aligned according to the aligner (−f 2) and with high mapping quality (−q 50). Replication timing profiles were generated by normalizing the replicating (S phase) sample to the nonreplicating (G2) sample in 1-kb windows. The *Saccharomyces castellii* profile was generated from the replicating sample only. Windows in which fewer than 250 (500 for *Tetrapisispora blattae*) reads were mapped in either sample were excluded. The resulting absolute ratios reflect the read numbers; therefore, data were normalized by dividing by an empirically determined factor. Data points <0.9 or >2.1 were excluded. Smoothing was applied using a Fourier transformation (custom Python script fft.py; Müller et al., 2014). Windows lacking data points for more than 5 kb were excluded from smoothing. All high-throughput sequencing data are deposited with NCBI under accession no. GSE89337.

### Assigning replication timing values to genetic elements

The position of every genetic element in each of the seven species as well as the ancestral relationship between elements were obtained from the Yeast Gene Order Browser (http://ygob.ucd.ie; Gordon et al., 2009). Smoothed replication timing data for each species provided a replication timing value for every base pair genome-wide. The replication timing value closest to the midpoint of each genetic element was then determined using “closestBed −d.” The cross-species mean replication time as well as the SD for every ancestral element was calculated.

### Random data simulations

The observed data are 4,616 ancestral elements with replication timing values from up to seven species. Data simulations were performed by randomly reassigning the measured replication times of all elements within each species (using the R command “sample”). In each simulation, the cross-species mean replication time and SDs were calculated for every ancestral element. 3,000 simulations were run, giving a total of 3,000 × 4,616 (13,848,000) values for both mean replication times and SDs. These values were analyzed using a 2D kernel density estimation (R package “kde2d”) to identify the thresholds encompassing 95, 99, and 99.9% of the simulated data (Fig. 2 A). The equation describing the line along the lower edge of the area representing 99% of simulated data was determined using Matlab’s “cftool.”

### Statistical analyses

Two-sample *z* tests with two-tailed comparisons were used to calculate the significance of the enrichment of functional annotations within the 221 elements with conserved replication time (Fig. 2 A). The statistical significance of the difference in histone mRNA abundance (Fig. 3 C) was calculated using two-tailed *t* tests.

### Identification of centromere-linked genetic elements

C-terminally tagging Dbf4 specifically abrogates its recruitment to centromeres (Natsume et al., 2013). The published study determined the probability of a difference in replication timing value between the wild-type and Dbf4-myc strains. These probabilities were assessed for each of the 221 *S. cerevisiae* elements with conserved replication timing. If the probability was 0.005% or less, the conservation of the respective genetic element was classified as centromere dependent.

### Time-course experiments

Cells were grown, arrested with α factor, and released at 23°C. Samples were collected every 2.5 min for flow cytometry analysis and every 5 min for isolation of mRNA. Samples for mRNA extraction were washed in water, resuspended in 400 µl TES buffer (10 mM Tris, pH 7.5, 10 mM EDTA, and 0.5% SDS) to which 400 µl acid phenol was added. Samples were vortexed for 10 s and incubated at 65°C for 60 min with vortexing every 15 min. The samples were placed on ice for 5 min and spun (14,000 rpm, 10 min, 4°C), and the aqueous phase was recovered. RNA was recovered by ethanol precipitation and resuspended in 100% formamide. RNA concentrations were measured by Nanodrop and cDNA synthesized from 1 µg RNA (ProtoScript II First Strand cDNA Synthesis kit; New England Biolabs, Inc.) using a d(T)_23_ primer (*S. cerevisiae* histone gene transcripts have a poly-A tail). Transcript abundance was determined by quantitative PCR using the SYBR Green JumpStart Taq ReadyMix (Sigma-Aldrich) and primers listed in Table S4.

### Analysis of transcript levels in an *rtt109*-deletion strain

Voichek et al. (2016) measured transcript levels at time points throughout S phase in synchronized cultures of wild-type and *rtt109Δ* cells. The authors provided their data as log2 values. Transcript levels were then calculated using this formula: 2*^tx^*^−^*^t0^*, i.e., 2 to the power of the difference between the arrested sample and the time points after release (Fig. 3 D).

### Online supplemental material

Fig. S1 provides additional information relevant to the replication timing comparisons between species. Fig. S1 A confirms that *S. cerevisiae* ohnologs do not have similar replication times. Fig. S1 B shows the distribution of the number of replication timing values for each ancestral element. Fig. S1 C shows the distribution of the number of elements that were below the threshold in simulated data. Fig. S1 D demonstrates that there is low conservation in replication time of genetic elements adjacent to conserved elements. Fig. S1 E shows that the majority of conserved elements are in single-gene clusters. Fig. S1 F shows that there is a statistically significant bias for codirectional replication and transcription of *S. cerevisiae* tRNA genes. Figs. S2 and S3 show the abundance of transcript levels of control genes and core histone genes at different times throughout a synchronous S phase, extending Fig. 3. Fig. S2 A shows the increase in bulk DNA content as synchronized wild-type and origin mutant cells progress through S phase. Fig. S2 (B–D) show transcript levels of control genes and core histone genes in wild-type and origin mutant strains. Fig. S3 further extends Fig. 3 by showing transcript levels in a biological repeat. Table S1 lists high-throughput sequencing samples from this study, number of mapped reads, and mapped reads/kb. Table S2 lists the 185 ancestral elements with conserved replication time and provides the normalized relative copy number for each species. Table S3 A lists the 221 *S. cerevisiae* genes with conserved replication timing, and Table S3 B, the gene ontology terms that are significantly enriched among them. Table S4 lists the sequences of primers used for this study. A custom Python Script, fft.py (Müller et al., 2014), was used to smooth replication timing data by truncating the Fourier transformation.

## Supplementary Material

Supplemental Materials (PDF)

Tables S1-S4 (zipped Excel files)

Data set S1 (.txt file)
