# Supplementary material for: DNA replication timing influences gene expression level
Source: J Cell Biol. 2017 Jul 3;216(7):1907–14. doi: 10.1083/jcb.201701061 (PMC5496624; doi:10.1083/jcb.201701061)
Supplement: Supplemental Materials (PDF) [file JCB_201701061_sm.pdf]

Müller and Nieduszynski, https://doi.org/10.1083/jcb.201701061

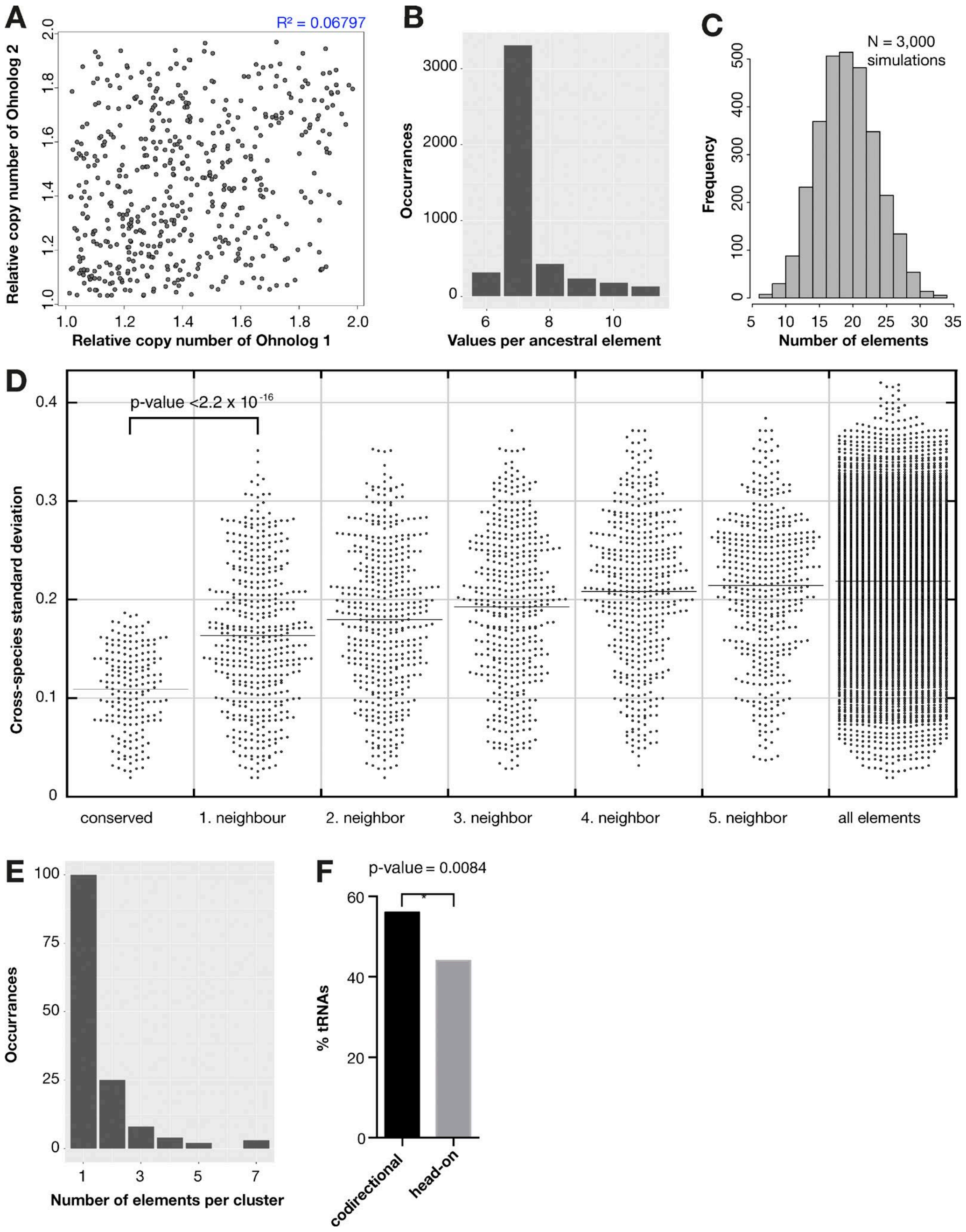

Figure S1. **Analysis of genomic features with conserved replication time.** (A) Comparison of replication times of *S. cerevisiae* ohnologs. The relative copy number of the two genes of every ohnolog was extracted from genome-wide replication timing data. The two corresponding values were plotted against each other. Conservation in replication times between ohnologs would result in data points forming a line. The R^2 value of 0.06797 indicates no conservation in replication time. (B) Bar graph showing the number of observed replication timing values for 4,616 ancestral elements. Our comparative genomics analysis included seven yeast species, four of which diverged after the WGD event. Therefore, ancestral elements could be associated with up to 11 replication timing values. Ancestral elements with fewer than six values were excluded from the analysis. (C) Histogram showing the number of elements in each of the simulations that were below the threshold demarcating conservation in replication timing. (D) Distribution of the SDs of replication timing values across species for conserved elements, their neighboring elements and all 4,616 elements (statistical significance was calculated using the two-sample Kolmogorov–Smirnov test). (E) Distribution of the number of elements per cluster. *S. cerevisiae* elements with conserved replication time were grouped into the same cluster when located adjacently in the genome. The histogram shows the number of elements in each of the clusters. (F) Bar graph showing the percentage of tRNA genes that are transcriptionally oriented codirectionally or head-on to replication (statistical significance was calculated using the probability mass function of a binomial distribution). Predominant replication fork direction was determined using published Okazaki fragment mapping data (Smith and Whitehouse, 2012).

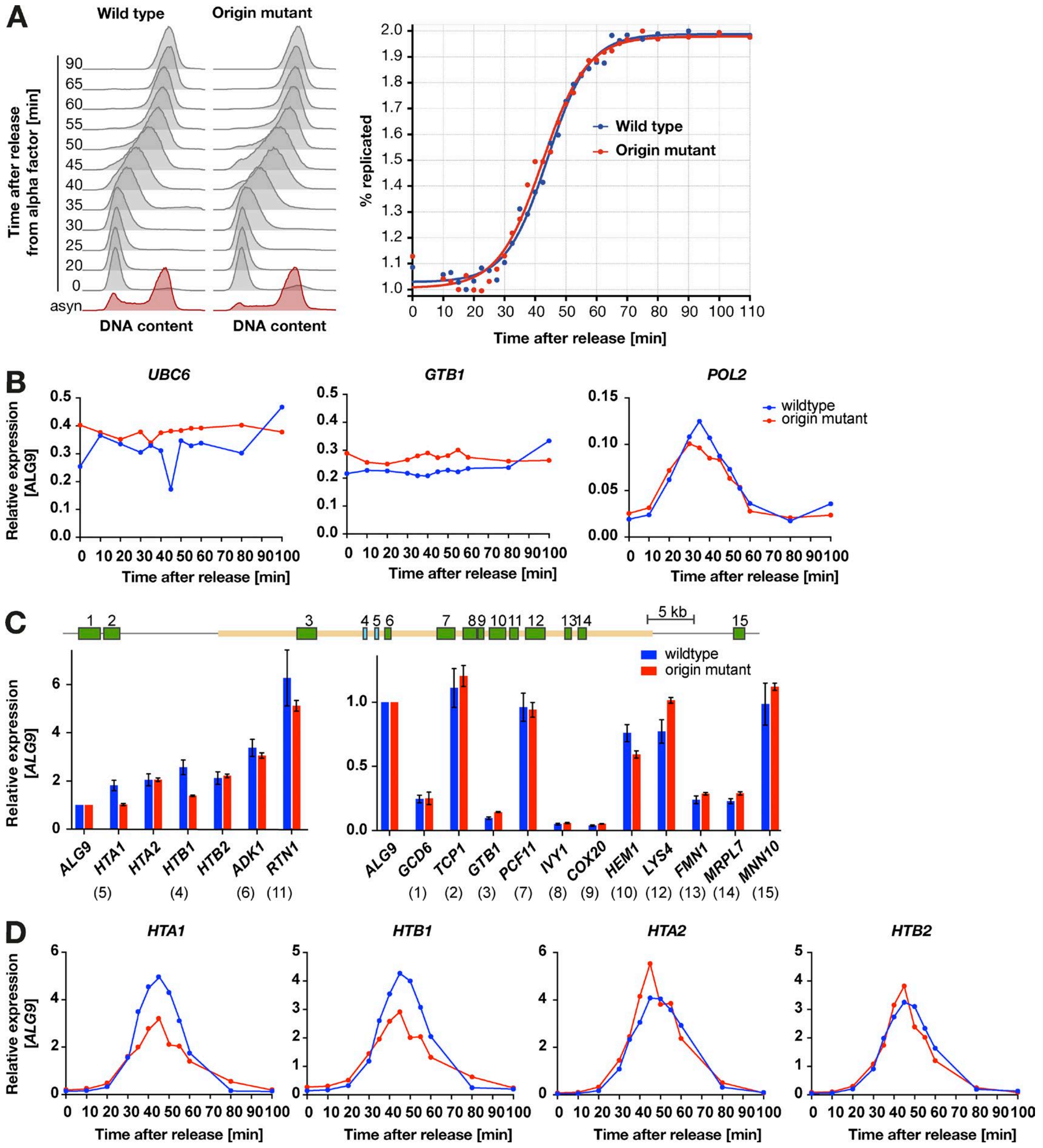

Figure S2. **RT-qPCR analysis of gene transcript levels in synchronized cultures of wild-type and origin mutant strains.** (A) Flow cytometry analysis of the DNA content of wild-type and origin mutant cultures released synchronously from α factor arrest (left; subset of time points visualized). Quantification of the flow cytometry data (with curve fit; right). RT-qPCR analysis in B–D was performed on samples taken during the time courses. (B) Time course of *UBC6*, *GTB1*, and *POL2* gene transcript levels. (C) Transcript levels of histone genes and control genes proximal to *HTA1-HBT1* 50 min after release from α factor (72% genome replication; independent cDNA synthesis from B and D). The cartoon indicates the location of analyzed genes (green boxes) and the region significantly ($P \leq 0.001$) delayed by origin inactivations (orange bar). Error bars represent ± SD between five technical repeats. (D) Time-course analysis of *HTA1*, *HTA2*, *HTB1*, and *HTB2* gene transcript levels in synchronized cultures of wild-type and the origin mutant strain (same cDNA as in B).

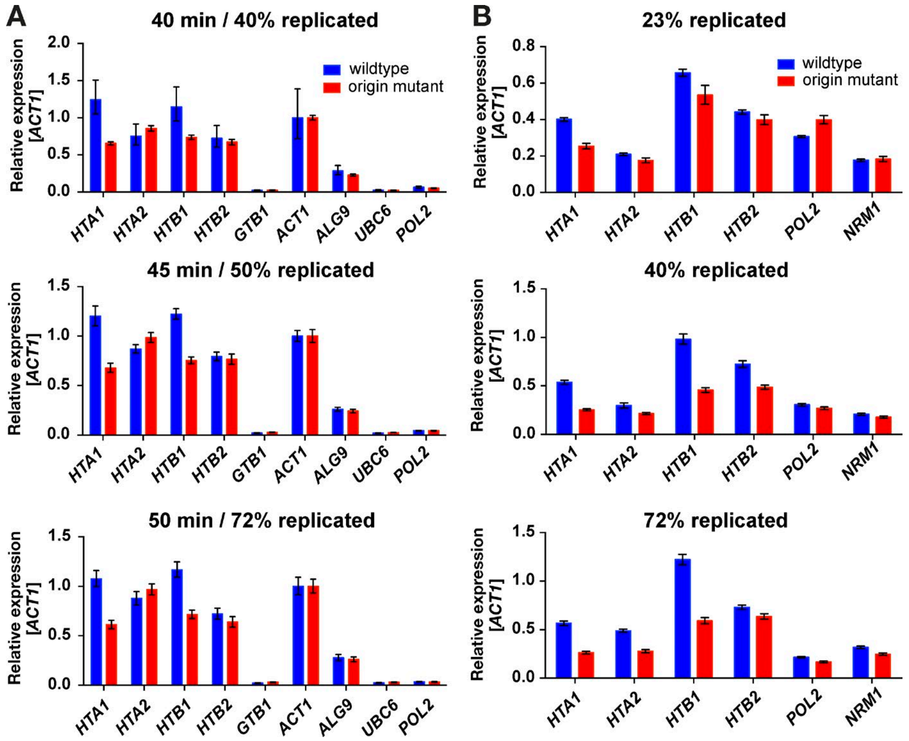

Figure S3. **Biological repeats of RT-qPCR analysis of gene transcript levels.** Comparison of two biological repeats of transcript-level quantifications through S phase for wild-type and origin mutant strains. Error bars represent ± SD between five (A) and nine (B) technical repeats.

Provided online are four Excel tables and one dataset. Table S1 lists high-throughput sequencing samples from this study, number of mapped reads, and mapped reads/kb. Table S2 lists the 185 ancestral elements with conserved replication time and provides the normalized relative copy number for each species. Table S3 A lists the 221 *S. cerevisiae* genes with conserved replication timing, and Table S3 B, the gene ontology terms that are significantly enriched among them. Table S4 lists the sequences of primers used for this study. A custom Python Script, fft.py (Müller et al., 2014), was used to smooth replication timing data by truncating the Fourier transformation.

References

Müller, C.A., M. Hawkins, R. Retkute, S. Malla, R. Wilson, M.J. Blythe, R. Nakato, M. Komata, K. Shirahige, A.P. de Moura, and C.A. Nieduszynski. 2014. The dynamics of genome replication using deep sequencing. *Nucleic Acids Res.* 42:e3. http://dx.doi.org/10.1093/nar/gkt878

Smith, D.J., and I. Whitehouse. 2012. Intrinsic coupling of lagging-strand synthesis to chromatin assembly. *Nature.* 483:434–438. http://dx.doi.org/10.1038/nature10895
